# Field Effectiveness of Pandemic and 2009-2010 Seasonal Vaccines against 2009-2010 A(H1N1) Influenza: Estimations from Surveillance Data in France

**DOI:** 10.1371/journal.pone.0019621

**Published:** 2011-05-10

**Authors:** Camille Pelat, Alessandra Falchi, Fabrice Carrat, Anne Mosnier, Isabelle Bonmarin, Clément Turbelin, Sophie Vaux, Sylvie van der Werf, Jean Marie Cohen, Bruno Lina, Thierry Blanchon, Thomas Hanslik

**Affiliations:** 1 UPMC Université Paris 06, UMRS 707, Paris, France; 2 U707, INSERM, Paris, France; 3 Unité de Santé Publique, Hôpital Saint-Antoine, Assistance Publique-Hôpitaux de Paris, Paris, France; 4 Réseau des GROG, Groupes Régionaux d'Observation de la Grippe, Paris, France; 5 Institut de Veille Sanitaire, Saint-Maurice, France; 6 Unit of Molecular Genetics of RNA Viruses, National Influenza Center (Northern-France), Department of Virology, Institut Pasteur, Paris, France; 7 URA3015, CNRS, Paris, France; 8 Université Paris Diderot, Sorbonne Paris Cité, Paris, France; 9 Laboratoire de Virologie, Centre de Biologie et de Pathologie Est, Hospices Civils de Lyon, CNR des Virus Influenzae - Région Sud, Bron, France; 10 Service de Médecine Interne, Hôpital Ambroise-Paré, Assistance Publique-Hôpitaux de Paris, Boulogne Billancourt, France; 11 Université Versailles-Saint-Quentin-en-Yvelines, Versailles, France; University of Hong Kong, Hong Kong

## Abstract

**Background:**

In this study, we assess how effective pandemic and trivalent 2009-2010 seasonal vaccines were in preventing influenza-like illness (ILI) during the 2009 A(H1N1) pandemic in France. We also compare vaccine effectiveness against ILI versus laboratory-confirmed pandemic A(H1N1) influenza, and assess the possible bias caused by using non-specific endpoints and observational data.

**Methodology and Principal Findings:**

We estimated vaccine effectiveness by using the following formula: VE  =  (PPV-PCV)/(PPV(1-PCV)) × 100%, where PPV is the proportion vaccinated in the population and PCV the proportion of vaccinated influenza cases. People were considered vaccinated three weeks after receiving a dose of vaccine. ILI and pandemic A(H1N1) laboratory-confirmed cases were obtained from two surveillance networks of general practitioners. During the epidemic, 99.7% of influenza isolates were pandemic A(H1N1). Pandemic and seasonal vaccine uptakes in the population were obtained from the National Health Insurance database and by telephonic surveys, respectively. Effectiveness estimates were adjusted by age and week. The presence of residual biases was explored by calculating vaccine effectiveness after the influenza period. The effectiveness of pandemic vaccines in preventing ILI was 52% (95% confidence interval: 30–69) during the pandemic and 33% (4–55) after. It was 86% (56–98) against confirmed influenza. The effectiveness of seasonal vaccines against ILI was 61% (56–66) during the pandemic and 19% (−10–41) after. It was 60% (41–74) against confirmed influenza.

**Conclusions:**

The effectiveness of pandemic vaccines in preventing confirmed pandemic A(H1N1) influenza on the field was high, consistently with published findings. It was significantly lower against ILI. This is unsurprising since not all ILI cases are caused by influenza. Trivalent 2009-2010 seasonal vaccines had a statistically significant effectiveness in preventing ILI and confirmed pandemic influenza, but were not better in preventing confirmed pandemic influenza than in preventing ILI. This lack of difference might be indicative of selection bias.

## Introduction

Estimating the field effectiveness of influenza vaccines (VE) poses specific challenges for the 2009 A(H1N1) pandemic. In particular, both pandemic and seasonal vaccination campaigns took place during the epidemic and, as a consequence, vaccine coverage changed through time, both in influenza cases and in the population as a whole.

**Figure 1:**
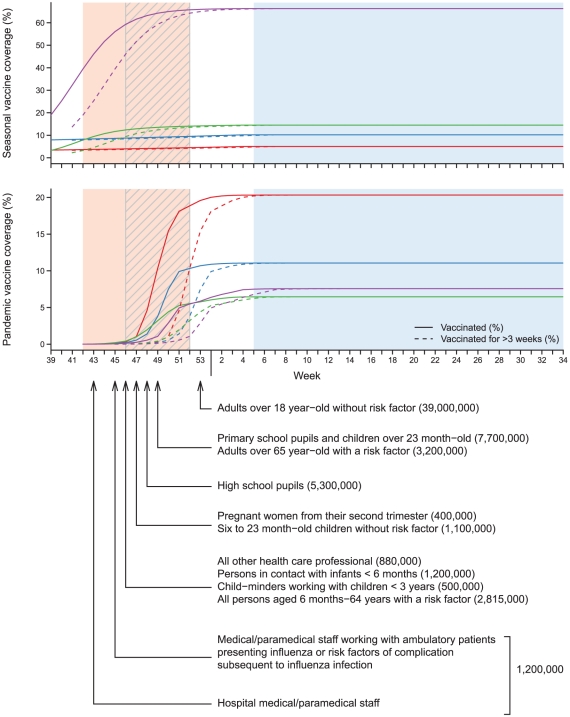
Weekly coverage of pandemic and seasonal vaccines in the population throughout the study period. Red curves: vaccine coverage in the 6 month- to 4 year-old age group; Blue: in the 5 to 14 year-old age group; Green: in the 15 to 64 year-old age group; Violet: in the ≥65 year-old age group. Pandemic vaccination targeted different at-risk groups, which were called in turn, according to a calendar established by French public health authorities. The principal steps of this calendar are outlined below the figure. Grey hatched area: epidemic study period for the effectiveness of pandemic vaccines (weeks 46/2009 to 52/2009); Pink area: epidemic study period for seasonal vaccines (weeks 42/2009 to 52/2009); Blue area: post-epidemic study period for pandemic and seasonal vaccines (weeks 05/2010 to 34/2010).

In France, pandemic vaccination conformed to a priority list established by public health authorities based on exposure and/or transmission probability, or on risk of complication subsequent to influenza [1]. The priority allocation of pandemic vaccines is shown in Figure 1, along with the evolution of vaccine coverage over time, by broad age categories. Medical and paramedical staffs working in hospitals were first called, on October 20^th^ (week 43). Individuals working with ambulatory patients presenting with influenza or working with patients at high risk of complication for influenza were called on November 2^nd^ (week 45). Risk factors of complication, stated in a High Committee of Public Health advice, on September 7^th^ 2009, were: pregnancy (in particular from the second trimester), obesity, and chronic conditions such as bronco-pulmonary diseases, heart diseases, diabetes and immunosuppression [2]. On November 12^th^ (week 46) all other health care professional were called (880,000), as well as all persons in contact with infants younger than six month-old (1,200,000), child-minders working with children under three year-old (500,000), and every person between six months and 64 years of age with a risk factor (2,815,000). Pregnant women from their second trimester and 6- to 23 month-old children without risk factor were called on November 20^th^ (week 47). High school pupils were called on November 25^th^ (week 48). People over 65 year-old with a risk factor (3,200,000) and children older than 23 month-old (7,700,000) and were called in week 49. Finally, adults over 18 year-old without a risk factor were called in week 53 (39,000,000). In the end, 63,295,000 persons had been called to vaccination centers to receive a pandemic influenza vaccine: all the French population, except infants younger than 6 month-old.

Seasonal vaccines were available to everyone at high risk of seasonal influenza related complications, from September 21^st^ (week 39/2009). For both vaccines, most vaccinations were completed by the end of January 2010.

**Figure 2:**
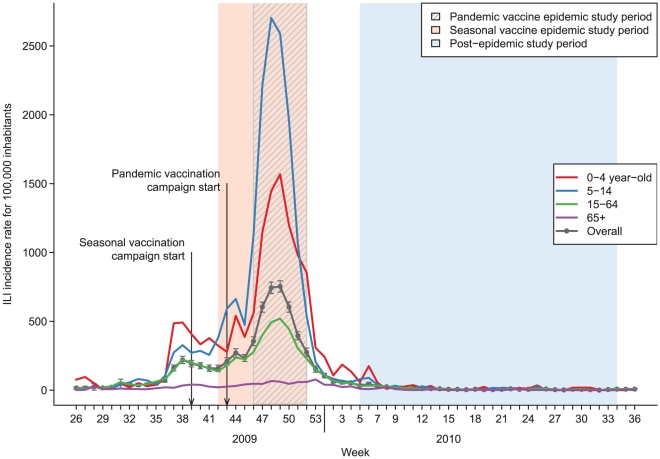
Weekly ILI incidence rates during the 2009–2010 pandemic in France (including Corsica, excluding overseas territories). Black curve: national ILI incidence rate and 95% confidence interval. Red curve: national ILI incidence rate in the 0 to 4 year-old age group; Blue: in the 5 to 14 year-old age group; Green: in the 15 to 64 year-old age group; Violet: in the ≥65 year-old age group.

In France, influenza-like illness (ILI) incidence has been monitored since 1984 by the *Sentinelles* network, a surveillance system based on sentinel general practitioners (GPs) [3]. ILI incidence crossed the epidemic threshold in week 37/2009, while detection of 2009 pandemic A(H1N1) viruses remained sporadic until week 42 (see Figure 2). ILI incidence peaked in week 49 and fell below the epidemic threshold in week 53. During the epidemic, the pandemic strain was dominant (99.7%) among influenza virus isolates [4]. In the first weeks of the pandemic, the sharpest increase in ILI incidence was observed in children under five year-old. After five weeks, 5 to 14 year-old children became the most affected group. The biggest difference between age-specific incidence rates was observed at the peak of the epidemic. It can be seen in Figure 2 that adults over 65 year-old were almost unaffected by the pandemic wave in France.

The purpose of this study was to assess how effective the pandemic and 2009-2010 trivalent seasonal vaccines were in preventing ILI on the field during the 2009-2010 season, using surveillance data. To evaluate how using ILI instead of a more specific influenza endpoint biased VE estimates, we conducted a validation study on a sample of laboratory-confirmed A(H1N1) influenza. We also assessed the existence of selection biases by estimating VE outside the influenza-circulating period.

## Methods

The Orenstein's screening method was used to calculate vaccine effectiveness with the following formula:



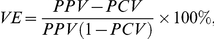
where *PCV* is the proportion of vaccinated among influenza cases and *PPV* is the proportion of vaccinated among the population [5], [6]. PPV was obtained from administrative sources. Two influenza datasets were used: one of clinically defined ILI cases, and one of laboratory-confirmed pandemic A(H1N1) influenza cases.

One injection was considered sufficient to confer a vaccinated status [7], [8]. Because influenza vaccines were not given to children under six month-old, they were excluded from the study. Individuals with missing age or vaccination status were also excluded.

### Study period

For ILI data, an epidemic and a post-epidemic study period were defined for each vaccine. The epidemic study period began three weeks after the start of the vaccination campaign: in week 42/2009 (October 12^th^) for seasonal vaccines and in week 46/2009 (November 9^th^) for pandemic vaccines, and lasted until week 52/2009, end of the epidemic. The post-epidemic study period started in week 05/2010 (February 1^st^) and lasted until week 34/2010 (August 29^th^). Weeks 53/2009 to 04/2010 constituted a “washout period” since residual circulation of influenza viruses was observed even though the epidemic itself was over. Study periods are shown in Figure 1 and 2.

For laboratory-confirmed data, a single study period, spanning weeks 46/2009 to 04/2010, was defined.

### Case recruitment

ILI cases were reported by *Sentinelles* GPs in France excluding overseas territories but including Corsica, as part of a surveillance routine using the following definition: “sudden onset of fever >39°C (>102°F) with respiratory signs and myalgia” [3].

Laboratory-confirmed influenza cases in France excluding Corsica and overseas territories were reported by physicians from the *Regional Groups of Influenza Observation (GROG)* as part of a surveillance routine. *GROG* is a network of private-practice GP and pediatricians dedicated to the virological surveillance of influenza [9]. Corsican laboratory-confirmed influenza cases were reported by *Sentinelles* GPs [10]. Nasopharyngeal swabs were collected through a randomized selection routine. Doctors included the first patient of each week, of any age (*Sentinelles* protocol) or of a personally assigned age group among 0–4, 5–14, 15–64, and ≥65 year-old (*GROG* protocol). Only patients presenting between 0 and 7 days after symptom onset were swabbed.

*GROG* doctors swabbed patients presenting with acute respiratory infection, defined as: sudden onset of a respiratory sign (cough, rhinitis, coryza,…) and a systemic sign evoking an infection (fever, asthenia, headache, myalgia, faintness). Corsican *Sentinelles* GPs swabbed patients presenting with ILI according to the *Sentinelles* definition. Swabs were analyzed in 11 laboratories, depending on the region of swabbing (two national reference centers and nine laboratories), by real-time polymerase chain reaction and/or culture.

For both ILI and laboratory-confirmed influenza cases, the following information was included: date of consultation, age, status regarding pandemic and seasonal influenza vaccination, and a logical indicating whether the time from vaccination to consultation was ≤3 or >3 weeks. The viral strain was known for laboratory-confirmed influenza cases.

### Population data

The pandemic vaccine coverage in the population was calculated from the National Health Insurance database (Caisse Nationale d'Assurance Maladie). The provided database contained weekly counts of all persons vaccinated in France (excluding overseas territories and including Corsica) with a pandemic vaccine, from the start of the vaccination campaign to week 18 of 2010. Detailed coverage was provided for nine age groups: 6 month- to 4 year-old, 5–14, 15–24, 25–34, 35–49, 50–64, 65–69, 70–74, and ≥75 year-old. Counts from weeks 19 to 34 of 2010 were assumed to be equal to those during week 18, since the data show that very few pandemic vaccinations were completed after winter.

Pandemic vaccine coverage at week *w*, for age group *g*, 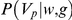, was calculated as:



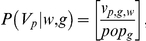
with 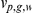 the number of people in age group *g* vaccinated with the pandemic vaccine before or during week *w*, and pop*_g_* the population size of age group *g* in 2009 in France (with Corsica, without overseas territories).

This vaccine coverage was monotonically increasing since 1) once vaccinated, people remained as such throughout the season and 2) the database contained all people vaccinated with a pandemic vaccine, so that no random fluctuation was present.

For seasonal vaccines, a similar database was not maintained by the National Health Insurance. Instead, vaccine coverage in the population was estimated each month from September 2009 to April 2010 by the Health Surveillance Institute (Institut de Veille Sanitaire, InVS) using computer-assisted telephone surveys. Each month a random sample from the French population (excluding overseas territories and including Corsica) were interviewed during an, on average, 11 day window. During the first stage of each survey, the sampling frame was the telephone numbers list, stratified by region and town size. At the second stage, the sampling frame was household residents, stratified by age (<5 years, ≥5 years). Each month around 800 questionnaires were filled in. Details of the sample design are provided elsewhere [11]. Pandemic vaccination status was also asked from November 2009, enabling identification of the proportion of people vaccinated with both vaccines, 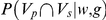.

For the purpose of calculating the weekly seasonal vaccine coverage, we began by attributing the interview data to the weeks that accounted for most days of the interview windows. In other words, using the interview data, we first estimated the proportion of people vaccinated with seasonal vaccines, 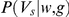, and co-vaccinated with pandemic and seasonal vaccines, 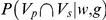, for weeks 38, 42, 47 and 50 of 2009, and 2, 7, 11 and15 of 2010.

We then fitted logistic functions to these data points to obtain smoothed, monotonically increasing weekly predictions of vaccine coverage for all weeks in the study period: 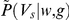 and 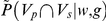. To account for the end of the vaccination campaigns, the predicted coverage of seasonal vaccines and of co-vaccinations, were constrained to remain stable respectively from week 5 and week 11 of 2010.

Finally, the weekly coverage of seasonal vaccines in people that did not get the pandemic vaccine, 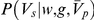, was calculated as:



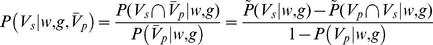



### Estimation of field vaccine effectiveness

We used Farrington's implementation of the screening method to estimate VE adjusted on age and time [12]. In brief, VE was estimated with a logistic regression model allowing a different offset in each age × time strata. Age was divided into nine strata (6 month- to 4 year-old, 5–14, 15–24, 25–34, 35–49, 50–64, 65–69, 70–74 and ≥75 year-old), and time was divided into one week strata.

A consensus view is that a period of at least 14 days is needed to achieve a protective concentration of antibody after influenza vaccination, but this timeline may vary [13]. Herein, for pandemic and seasonal influenza vaccines, individuals were considered vaccinated three weeks after receiving a dose of vaccine, and unvaccinated if they had received no vaccine or if the vaccine had been given in the last three weeks.

In practice, for the calculation of VE in a week t, we compared the proportion of population vaccinated for more than three weeks at week t to the proportion of influenza cases vaccinated for more than three weeks that were seen by sentinel GPs in week t. Figure 1 shows the proportion of vaccinated in the population (plain lines), and the proportion of population that has been vaccinated for more than three weeks (dashed lines).

Since delay from injection was missing for some influenza cases, we ran two analyses. In the first one we assumed that these cases had received influenza vaccination in the three weeks preceding the consultation, and included them as unvaccinated. In the second analysis, we assumed that they had been vaccinated for more than three weeks and we included them as vaccinated. We explored in a third analysis what vaccine effectiveness was when vaccination was considered completed immediately after the injection of one vaccine dose. Because the question related to the interval between consultation and vaccination was dichotomized at three weeks, we could no further explore the protection timeline.

As trivalent 2009–2010 seasonal vaccines were expected to be less specific of 2009 A(H1N1) than pandemic vaccines, effectiveness of the seasonal vaccines was calculated only using those individuals that did not receive pandemic vaccination. As a sensitivity analysis, a second analysis included those cases.

### Assessment of bias

The use of ILI, a non-specific influenza outcome, as a primary endpoint for estimating the effectiveness of influenza vaccines can bias VE estimates downward. Indeed, if only 50% of ILI cases are caused by influenza, a hundred percent effective influenza vaccine will have at most 50% effectiveness in preventing ILI: as the specificity of the outcome decreases, so do VE estimates [14]–[16]. To assess this first source of bias, we compared VE estimates obtained using the ILI case sample provided by *Sentinelles* GPs to VE estimates obtained using the confirmed pandemic A(H1N1) influenza cases provided by *GROG* and some *Sentinelles* GPs.

A second source of bias was identified in that we used observational data of patients consulting a GP for an influenza episode, and compared their vaccine coverage to the one in the general population. Patients consulting a GP for an influenza episode might not be comparable to the rest of the population. In particular, if people vaccinated with influenza vaccine consulted differently than the rest of the population for an influenza episode, because of comorbidities or because of a different propensity to seek care for example, this could be a confusion factor when estimating VE. As suggested by several authors, such a selection bias should affect VE estimated on GP data during the influenza-circulation period as well as VE estimated outside this period [17]–[22]. This is particularly interesting when using ILI, as influenza vaccine effectiveness in preventing ILI should be null outside the influenza-circulation period, if no bias was present. We therefore calculated VE against ILI after the influenza epidemic period, as an indication of selection bias in our data. As no vaccine was given before the epidemic start, pre-epidemic VE could not be assessed. We did not calculate VE against laboratory-confirmed A(H1N1) influenza outside the influenza-circulating period.

## Results

### ILI cases

#### Pandemic vaccines

During the study period, *Sentinelles* GPs reported 7586 ILI cases above 6 month-old, with information on age and pandemic vaccine status, which were included in the pandemic vaccines analysis. Among them, 172 (2%) were vaccinated. Detailed numbers by study period and age group are presented in Table 1.

**Table 1:** Pandemic vaccine uptake among ILI cases included in the pandemic vaccine effectiveness analysis.

	*Epidemic study period*					*Post-epidemic study period*				
			Vaccinated for					Vaccinated for		
Age group	Total described	Total vaccinated	>3 weeks	Unknown	≤3 weeks	Total described	Total vaccinated	>3 weeks	Unknown	≤3 weeks
6 m–4 y	875	43	13	1	29	51	5	4	1	0
5–14	2740	44	6	4	34	89	9	7	0	2
15–24	1250	13	1	2	10	55	2	2	0	0
25–34	763	7	1	0	6	80	3	3	0	0
35–49	937	19	2	0	17	135	11	11	0	0
50–64	378	8	1	0	7	68	6	5	0	1
65–69	46	0	0	0	0	11	0	0	0	0
70–74	28	2	0	0	2	10	0	0	0	0
≥75	55	0	0	0	0	15	0	0	0	0
**Total**	**7072**	**136**	**24**	**7**	**105**	**514**	**36**	**32**	**1**	**3**

Pandemic influenza vaccination had been done in the three weeks preceding the consultation for 108 vaccinated cases (63%), more than three weeks before consultation for 56 cases (33%), or at an unknown date for 8 of them. During the pandemic period, 67% (29/43) of the 6 month- to 4 year-old ILI cases were vaccinated in the three weeks preceding the consultation, versus 77% (34/44) of the 5 to14 year-old and 86% (42/49) of the 15 to 64 year-old (Fisher's test p = 0.12). None of the two vaccinated ILI cases ≥65 year-old reported by *Sentinelles* GPs during the pandemic had been vaccinated for more than three weeks.

Six month to four year-old children mostly received the non-adjuvanted Panenza® vaccine (21/48 i.e. 44%); four of them (8%) received the adjuvanted Pandemrix® vaccine; trademark was unknown for the remaining 23 ones. On the contrary, 5 to 14 year-old children mostly received the Pandemrix® vaccine (22/53 i.e. 42%); nine of them (17%) received the non-adjuvanted Panenza® vaccine; trademark was unknown for the remaining 22 ones. Most cases aged15 year-old and over received the Pandemrix® vaccine (43/71 i.e. 61%); four had the Panenza®, four the Q-Pan H1N1® and one the Focetria® vaccine; trademark was unknown for 19. Most cases received one dose of vaccine (154/172 i.e. 90%). Eight cases received two doses of vaccines: three children below 15 year-old and five 35 to 64 year-old adults.

#### Seasonal vaccines

For the purpose of estimating the effectiveness of seasonal influenza vaccines, 9564 cases were eligible. Among them, 540 (6%) were vaccinated. Detailed numbers by study period and age group are presented in Table 2. Vaccination had been done in the three weeks preceding the consultation for 115 cases (21%), more than three weeks before consultation for 401 cases (74%), or at an unknown date for 24 of them. Sixty-one of them (11%) were also vaccinated with the pandemic vaccine, while 465 (86%) were not, the remaining 14 patients having not answered the pandemic vaccination question.

**Table 2:** Seasonal vaccine uptake among ILI cases included in the seasonal vaccine effectiveness analysis.

	*Epidemic study period*					*Post-epidemic study period*				
			Vaccinated for					Vaccinated for		
Age group	Total described	Total vaccinated	>3 weeks	Unknown	≤3 weeks	Total described	Total vaccinated	>3 weeks	Unknown	≤3 weeks
6 m–4 y	1123	18	12	1	5	54	3	3	0	0
5–14	3447	73	43	2	28	100	4	4	0	0
15–24	1571	48	38	2	8	59	3	3	0	0
25–34	1020	34	21	4	9	83	6	5	0	1
35–49	1205	95	69	6	20	145	24	20	1	3
50–64	486	95	72	4	19	76	21	20	0	1
65–69	57	23	18	1	4	11	3	2	1	0
70–74	36	23	14	1	8	11	8	8	0	0
≥75	65	48	40	0	8	15	11	9	1	1
**Total**	**9010**	**457**	**327**	**21**	**109**	**554**	**83**	**74**	**3**	**6**

### Laboratory-confirmed cases

#### Pandemic vaccines

Together, the *GROG* and *Sentinelles* samples provided 838 laboratory-confirmed pandemic A(H1N1) influenza cases ≥6 month-old that were described for pandemic vaccination and age, and were included in the analysis of pandemic vaccines. We did not include in the analysis those influenza isolates that were not typed (18), were of not-subtyped type A (26), or were of subtype A(H3N2) (one). No B strain was present in the sample.

Twenty-five of the included A(H1N1) cases were vaccinated (3%). Detailed numbers by age group are presented in Table 3. It is noteworthy that none of the five confirmed A(H1N1) cases aged ≥65 year-old that were included in the study had received a pandemic vaccine.

**Table 3:** Pandemic vaccine uptake among laboratory-confirmed cases included in the pandemic vaccine effectiveness analysis.

			Vaccinated for		
Age group	Total described	Total vaccinated	>3 weeks	Unknown	≤3 weeks
6 m–4 y	209	13	0	2	11
5–14	339	9	1	0	8
15–24	93	1	1	0	0
25–34	78	0	0	0	0
35–49	85	0	0	0	0
50–64	29	2	0	1	1
65–69	2	0	0	0	0
70–74	0	0	0	0	0
≥75	3	0	0	0	0
**Total**	**838**	**25**	**2**	**3**	**20**

Six month- to four year-old children mostly received the non-adjuvanted Panenza® vaccine (12/13 i.e. 92%); only one of them received the adjuvanted Pandemrix® vaccine. Four of the nine vaccinated children between five and fourteen year-old were given the Panenza® vaccine (44%); three receive the Pandemrix® vaccine (33%); two received a pandemic vaccine of unknown brand. Among the three vaccinated cases above 15 years, two received the Pandemrix® and one the Panenza® vaccine.

Vaccination had been done in the three weeks preceding the consultation for 20 of the 25 vaccinated cases (80%), before that delay for two cases (8%), or at an unknown date for three of them (12%). Eighty-five percent (11/13) of the 6 month- to 4 year-old laboratory-confirmed cases were vaccinated in the three weeks preceding the consultation, versus 89% (8/9) of the 5 to14 year-old and 33% (1/3) of the 15 to 64 year-old (Fisher's test p = 0.16). Twenty-three cases (92%) had received one dose of vaccine, the other two cases did not precise this information. None has received two injections.

#### Seasonal vaccines

The laboratory-confirmed case sample contained 856 pandemic A(H1N1) cases that were described for age and seasonal vaccine status and could be included in the study. Fifty-one of them (6%) were vaccinated. Detailed numbers by age group are presented in Table 4.

**Table 4:** Seasonal vaccine uptake among laboratory-confirmed cases included in the seasonal vaccine effectiveness analysis.

			Vaccinated for		
Age group	Total described	Total vaccinated	>3 weeks	Unknown	≤3 weeks
6 m–4 y	213	5	2	0	3
5–14	342	17	11	2	4
15–24	98	4	3	0	1
25–34	82	3	0	2	1
35–49	86	7	4	3	0
50–64	30	10	5	4	1
65–69	2	2	1	1	0
70–74	0	0	0	0	0
≥75	3	3	3	0	0
**Total**	**856**	**51**	**29**	**12**	**10**

Vaccination had been done in the three weeks preceding the consultation for 10 of the vaccinated cases (20%), more than three weeks before consultation for 29 cases (57%), or at an unknown date for 12 of them (24%). All confirmed pandemic A(H1N1) cases ≥65 year-old included in the study had received the seasonal vaccine. Five (10%) of the cases vaccinated with a seasonal vaccine had also received a pandemic vaccine, while 43 (84%) had not.

### Field vaccine effectiveness

As exposed in the method section, three analyses were carried out. 1) In the first one (primary one), vaccination is considered completed three weeks after injection and all influenza cases with missing injection date are treated as unvaccinated. 2) As a first sensitivity analysis, influenza cases with missing vaccination date are treated as vaccinated. 3) Vaccination is considered completed immediately after the injection of one vaccine dose.

Since, during the pandemic, most reported influenza cases were vaccinated in the three weeks that preceded the consultation, considering missing injection date as belonging to the last three weeks, as was done in the first analysis, might be the more realistic option. The first analysis is thus considered the primary one, and its main results are reported hereafter. Detailed results of the three analyses are provided in Table 5 and Table 6.

**Table 5:** Field effectiveness of pandemic vaccines, by case definition, age group and study period.

	Age group	FVE (%)	95% CI
***ILI, epidemic period***			
**Vaccinated for** ≤**3 weeks or at an unknown date are considered unvaccinated**	6 m–4 y	12	(−47–53)
	5–14	53	(1–82)
	15–64	77	(51–92)
	≥65	100	–
	**All ages**	**52**	**(30**–**69)**
**Vaccinated for** ≤**3 weeks are considered unvaccinated. Vaccinated at an unknown date are considered vaccinated.**	6 m–4 y	5	(−57–47)
	5–14	18	(−47–60)
	15–64	68	(38–86)
	≥65	100	–
	**All ages**	**38**	**(13**–**58)**
**All vaccinees are considered vaccinated, regardless of the injection date**	6 m–4 y	53	(36–66)
	5–14	59	(46–70)
	15–64	49	(33–62)
	≥65	30	(−124–88)
	**All ages**	**54**	**(45**–**61)**
***ILI, post-epidemic period***			
**Vaccinated for** ≤**3 weeks or at an unknown date are considered unvaccinated**	6 m–4 y	65	(14–90)
	5–14	28	(−45–70)
	15–64	11	(−38–47)
	≥65	100	–
	**All ages**	**33**	**(4**–**55)**
**Vaccinated for** ≤**3 weeks are considered unvaccinated. Vaccinated at an unknown date are considered vaccinated.**	6 m–4 y	56	(−2–85)
	5–14	28	(−45–70)
	15–64	11	(−38–47)
	≥65	100	–
	**All ages**	**31**	**(1**–**53)**
**All vaccinees are considered vaccinated, regardless of the injection date**	6 m–4 y	56	(−2–85)
	5–14	5	(−78–56)
	15–64	6	(−44–43)
	≥65	100	–
	**All ages**	**24**	**(−7**–**47)**
***Laboratory-confirmed influenza***			
**Vaccinated for** ≤**3 weeks or at an unknown date are considered unvaccinated**	6 m–4 y	100	–
	5–14	72	(−28–98)
	15–64	74	(−15–99)
	**All ages**	**86**	**(56**–**98)**
**Vaccinated for** ≤**3 weeks are considered unvaccinated. Vaccinated at an unknown date are considered vaccinated.**	6 m–4 y	69	(1–95)
	5–14	72	(−28–98)
	15–64	48	(−64–91)
	**All ages**	**64**	**(21**–**87)**
**All vaccinees are considered vaccinated, regardless of the injection date**	6 m–4 y	44	(4–70)
	5–14	44	(−4–73)
	15–64	69	(19–92)
	**All ages**	**49**	**(25**–**67)**

**Table 6:** Field effectiveness of seasonal vaccines, by case definition, age group and study period.

		*Excluding pandemic vaccine recipients*		*Including pandemic vaccine recipients*	
	Age group	FVE (%)	95% CI	FVE (%)	95% CI
***ILI, epidemic period***					
**Vaccinated for** ≤**3 weeks or at an unknown date are considered unvaccinated**	6 m–4 y	77	(59–89)	72	(54–85)
	5–14	84	(78–89)	87	(83–91)
	15–64	52	(44–59)	47	(38–54)
	≥65	19	(−14–42)	21	(−10–44)
	**All ages**	**61**	**(56**–**66)**	**63**	**(58**–**67)**
**Vaccinated for** ≤**3 weeks are considered unvaccinated. Vaccinated at an unknown date are considered vaccinated.**	6 m–4 y	75	(56–87)	70	(51–84)
	5–14	83	(77–88)	87	(82–90)
	15–64	47	(39–55)	42	(33–50)
	≥65	14	(−20–38)	17	(−16–40)
	**All ages**	**58**	**(53**–**63)**	**60**	**(55**–**64)**
**All vaccinees are considered vaccinated, regardless of the injection date**	6 m–4 y	67	(44–82)	62	(42–77)
	5–14	69	(60–76)	79	(73–83)
	15–64	41	(33–49)	41	(33–48)
	≥65	−7	(−50–23)	−2	(−43–27)
	**All ages**	**47**	**(42**–**53)**	**54**	**(49**–**58)**
***ILI, post-epidemic period***					
**Vaccinated for** ≤**3 weeks or at an unknown date are considered unvaccinated**	6 m–4 y	54	(−112–97)	−18	(−220–71)
	5–14	61	(−22–94)	61	(7–88)
	15–64	7	(−32–37)	7	(−27–33)
	≥65	25	(−52–62)	39	(−23–70)
	**All ages**	**19**	**(−10**–**41)**	**19**	**(−5**–**39)**
**Vaccinated for** ≤**3 weeks are considered unvaccinated. Vaccinated at an unknown date are considered vaccinated.**	6 m–4 y	54	(−112–97)	−18	(−220–71)
	5–14	61	(−22–94)	61	(7–88)
	15–64	7	(−32–37)	4	(−30–31)
	≥65	3	(−99–52)	22	(−62–61)
	**All ages**	**14**	**(−16**–**38)**	**15**	**(−11**–**35)**
**All vaccinees are considered vaccinated, regardless of the injection date**	6 m–4 y	54	(−108–97)	−16	(−216–72)
	5–14	61	(−23–94)	62	(8–88)
	15–64	−4	(−47–28)	−7	(−44–22)
	≥65	−6	(−119–47)	17	(−72–59)
	**All ages**	**5**	**(−27**–**31)**	**7**	**(−21**–**29)**
***Laboratory-confirmed influenza***					
**Vaccinated for** ≤**3 weeks or at an unknown date are considered unvaccinated**	6 m–4 y	74	(18–96)	77	(29–96)
	5–14	60	(25–82)	65	(40–82)
	15–64	64	(36–82)	59	(29–78)
	**All ages**	**60**	**(41**–**74)**	**61**	**(44**–**74)**
**Vaccinated for** ≤**3 weeks are considered unvaccinated. Vaccinated at an unknown date are considered vaccinated.**	6 m–4 y	74	(18–96)	77	(29–96)
	5–14	50	(11–75)	59	(31–78)
	15–64	29	(−11–57)	24	(−16–53)
	**All ages**	**38**	**(14**–**57)**	**43**	**(23**–**60)**
**All vaccinees are considered vaccinated, regardless of the injection date**	6 m–4 y	31	(−51–75)	46	(−18–81)
	5–14	26	(−22–59)	48	(18–69)
	15–64	22	(−18–51)	25	(−12–52)
	**All ages**	**20**	**(−7**–**42)**	**35**	**(14**–**52)**

#### Effectiveness of pandemic vaccines

When vaccination was considered completed three weeks after the injection of one vaccine dose, the effectiveness of pandemic vaccine in preventing ILI was 52% (95% confidence interval 30–69) in the epidemic period, and 33% (4–55) in the post-epidemic period. When vaccination was considered completed immediately after injection, pandemic vaccine effectiveness in preventing ILI was 54% (45–61) in the epidemic period and 24% (−7–47) in the post-epidemic period.

When vaccination was considered completed three weeks after injection, pandemic vaccine effectiveness in preventing laboratory-confirmed pandemic A(H1N1) influenza was 86% (56–98). When vaccination was considered completed immediately after injection, pandemic vaccine effectiveness in preventing laboratory-confirmed pandemic A(H1N1) influenza was 49% (25–67).

The effectiveness of pandemic vaccines in preventing ILI and confirmed pandemic A(H1N1) influenza in different age groups is presented in Table 5. As no confirmed case above 65 year-old was vaccinated, the age-specific effectiveness of pandemic vaccine in this age group is not presented.

#### Effectiveness of seasonal vaccines

In the primary analysis of the field effectiveness of trivalent 2009–2010 seasonal vaccine, shown below, we excluded those individuals that received pandemic vaccination. The results of the analysis that included them are shown in the last column of Table 6.

When vaccination was considered completed three weeks after the injection of one vaccine dose, the effectiveness of seasonal vaccine in preventing ILI was 61% (56–66) in the epidemic period, and 19% (−10–41) in the post-epidemic period. When vaccination was considered completed immediately after injection, seasonal vaccine effectiveness in preventing ILI was 47% (42–53) in the epidemic period and 5% (−27–31) in the post-epidemic period.

When vaccination was considered completed three weeks after the injection of one vaccine dose, the effectiveness of seasonal vaccine in preventing laboratory-confirmed pandemic A(H1N1) influenza was 60% (41–74). When vaccination was considered completed immediately after injection, seasonal vaccine effectiveness in preventing laboratory-confirmed pandemic A(H1N1) influenza was 20% (−7–42).

The effectiveness of seasonal vaccines in preventing ILI and confirmed pandemic A(H1N1) influenza by age groups is presented in Table 6. Since only five laboratory-confirmed pandemic A(H1N1) cases older than 65 year-old were included in the analysis, we do not present age-specific VE for this age group.

## Discussion

We obtained estimates of vaccine effectiveness by comparing vaccine coverage of influenza cases (clinically defined or biologically confirmed) to vaccine coverage of population samples, using Orenstein's screening method [5],[12]. To allow for a delay between the injection of a vaccine dose and immunization, vaccination was considered completed only three weeks after injection. In other words, in our primary analysis, individuals vaccinated for less than three weeks were considered unvaccinated. As a consequence of the late vaccination campaigns, the number of vaccinations we could consider completed during the pandemic was low.

### Pandemic vaccine effectiveness

We found that pandemic vaccines had a field effectiveness of 86% (56–98) in preventing laboratory-confirmed pandemic A(H1N1) influenza, which is in the range of previously reported values [7], [8], [23]-[26]. More specifically, our estimates are slightly above the results of a multicenter case-control study conducted in seven European countries and are above the results of a case-control study conducted in the United Kingdom, which both found VE = 72% [8], [23]. They are also above the VE found by a Korean study, namely 73% [24]. However, they are below the estimates from a Scottish cohort study (95%) [25], a German study based on the screening method (83–97%) [26] and a Canadian test-negative incident case-control study (>90%) [7].

We found that pandemic vaccines had a significantly lower effectiveness in preventing ILI than in preventing confirmed A(H1N1) influenza: 52% (30–69) versus 86% (56–98). This is unsurprising as only a part of the 2009–2010 ILI cases were due to pandemic A(H1N1) influenza and therefore could be prevented by the pandemic vaccine. This part was estimated between 30% and 55%, depending on the country [27], [28].

The effectiveness of pandemic vaccines in preventing ILI and confirmed influenza seemed to vary with age, yet not significantly and in no reproducible order through our different analysis. Since few reported influenza cases were vaccinated, large confidence intervals surround our age-specific VE estimates. In particular, no confirmed A(H1N1) case and only two ILI cases above 65 year-old were vaccinated with the pandemic vaccine. Indeed, elderly people were not a priority target group for pandemic vaccination and were called late to vaccination centers (Figure 1). Estimations of vaccine effectiveness in this subgroup are therefore highly uncertain.

As others, we considered that one dose of pandemic vaccine was sufficient to provide immunization for all age classes [7], [8], [23], [24], [26]. However, it was shown that a single dose of pandemic A(H1N1) vaccine was more immunogenic in children older than three years than in younger children, and that a second dose was needed to reach seroprotection and seroconversion rates of 90–99% in both of these age groups [29].

Influenza vaccines are theoretically expected to be more effective three weeks after their injection than immediately after. We did observe a significantly higher effectiveness of pandemic vaccines in preventing laboratory-confirmed pandemic A(H1N1) influenza when only vaccinations dating back to more than three weeks were considered completed than when treating all vaccinations as completed: 86% (56–98) versus 49% (25–67). Yet, we did not find a significant difference in that respect regarding the effectiveness of pandemic vaccines against ILI: 52% (30–69) versus 54% (45–61), which is a first indication of the presence of biases in the estimation.

### Seasonal vaccine effectiveness

We found that the 2009–2010 seasonal vaccines had a significant effectiveness in preventing laboratory-confirmed influenza: VE = 60% (41–74). Unlike pandemic vaccines, seasonal vaccines were not significantly better in preventing laboratory-confirmed pandemic A(H1N1) influenza than in preventing ILI: respectively VE = 60% (41–74) versus VE = 61% (56–66). This result cannot be explained by ILI comprising an appreciable number of seasonal influenza cases against which seasonal vaccine could have proven effective: more than 99% of influenza isolates during the 2009-2010 season were pandemic A(H1N1) [4]. This result might, instead, indicate that our effectiveness estimates of seasonal vaccines are driven by biases, in particular case selection biases. Indeed, the population that yearly vaccinates against seasonal influenza might be distinct from the rest of the population, in particular in its propensity to consult. This could induce a selection bias, since influenza cases are recruited through GPs. Therefore, the absence of adjustments in our study, when most studies adjusted by comorbidities and previous vaccination, could be a reason for the high effectiveness we observed.

We identified a source of bias in our seasonal vaccine study in that we used underestimated seasonal vaccine coverage for the 6 month- to 4 year-old population age group. Indeed, the vaccine coverage data provided by the InVS study concerned the 0–4 year-old age group, even if 0 to 6 month-old children were not concerned by seasonal influenza vaccination. Nevertheless, this should result in under-estimating, not over-estimating, vaccine effectiveness in this age group.

Our results come within a succession of contradictory evidences regarding the effectiveness of seasonal vaccines against pandemic 2009 A(H1N1) influenza. In a literature review by Viboud et al, four studies of the 2008–2009 seasonal vaccines found no protection against 2009 pandemic A(H1N1) influenza; two other found a significant protection; two other found that 2008–2009 seasonal vaccination increased the risk of contracting pandemic 2009 A(H1N1) influenza [30]. A subsequent paper evidenced a moderate effectiveness of the 2008–2009 seasonal vaccines against pandemic A(H1N1) mild outcomes (VE = 42%, 29–53%) [31], while another one put forward an increased risk (odds-ratio = 2.45, 1.34–4.48) [32].

Regarding the effectiveness of the 2009–2010 seasonal vaccines, most works found no protection against confirmed pandemic A(H1N1) [8], [23],[33]–[36], while one found an effectiveness of 50% (40–59) in preventing pandemic A(H1N1) related hospitalizations [37].

### Adjustments

We adjusted by age to take into account different vaccine responses with age, and used weekly strata to account for the evolution of vaccine coverage in the population throughout the study period. As noted before, the lack of further adjustment could result in biased VE estimates, both for ILI and confirmed influenza. However, as our study was integrated in an ongoing surveillance system based on GP voluntary reporting of ILI cases, adjustment covariates were not collected in order to keep the questionnaire as short as possible. As a compromise, only essential covariates for VE calculation were asked to GPs: age, vaccine status (pandemic and seasonal), delay since vaccination (dichotomized at three weeks), and vaccine trademark (for pandemic vaccine only). Considering the small number of reported vaccinated influenza cases, pandemic VE was not calculated by vaccine trademark.

We used a three weeks delay between vaccination and consultation to differentiate vaccinated influenza cases fully immunized from those that were still unprotected. This delay might be too astringent: in most papers, a two week delay between vaccination and *symptom onset* is used [7], [23], [24], [26], as data on seasonal influenza showed that protective antibodies were present in over 90% of persons fourteen days after vaccination [13]. However, as the question used for ILI cases was “Is delay since vaccination ≤3 weeks?”, no data was available to make sensitivity analysis.

### Biases

#### Bias due to the use of a non-specific endpoint

ILI is a non-specific proxy for influenza infection. Disposing of a validation sample of laboratory-confirmed pandemic A(H1N1) influenza cases allowed us to explore the bias in VE inherent to the use of such a non-specific outcome. Regarding the effectiveness of pandemic vaccines, the results are in accordance with the theoretical principle which stipulates that, as the specificity of the outcome decreases, so should the measured vaccine effectiveness [14]–[16]. Effectiveness of seasonal vaccines, however, did not show such a difference.

A few studies have used ILI as an endpoint for evaluating influenza vaccine effectiveness on the field [38]–[41], although laboratory-confirmed influenza is a more common endpoint. In particular, a case-control study based on university students reporting ILI episodes through a web interface evidenced a significant reduction of ILI among vaccinated students during seasonal influenza (adjusted odds ratio: 0.70, 95% confidence interval 0.56–0.89) but not during non-influenza periods (adjusted odds ratio: 0.98, 0.73–1.30) [42].

Herein, we do observe a lower effectiveness of pandemic vaccines in preventing ILI after the epidemic period (VE = 33%, 4–55); however, unlike in Nichol et al, the effectiveness remains significantly above zero. As suggested by previous works, we can attribute this overestimation to the presence of biases, such as selection biases [17]–[22]. Stochastic variations due to our little sample sizes are another possible explanation.

Sensitivity of the outcome “ILI consulting a GP” to track true influenza is also an issue: asymptomatic cases, subclinical cases and presentations different from the chosen ILI definition are missed. Call et al reviewed the probabilities of presence of different symptoms in influenza cases, i.e. the sensitivity of these symptoms for detecting influenza: depending on the age group, they were between 0.3 and 0.9, with lower sensitivities in the elderly [43]. Orenstein et al demonstrated in a simulation study that poorly sensitive outcomes could lead to underestimation of field vaccine effectiveness in three different observational designs [16]. However, our analysis seems more subject to overestimation biases.

#### Bias due to incomparability of cases and population sample

Another potential source of bias in our study is the uncertain comparability of cases recruited by GPs with samples drawn from the general population. To further assess the possible lack of comparability between cases and population samples, an alternative population sample taken directly among GPs usual patients could be used for validation. To that effect, during 2010–2011 influenza season, a cross-sectional survey at GPs offices will be carried out to assess the vaccine coverage among *Sentinelles* GPs patients.

#### Selection biases

As was visible in the results of seasonal vaccine effectiveness, selection biases might be present in our study design and bias result upwards. To try and further evidence these selection biases, vaccine effectiveness against ILI was computed after the influenza circulation period. Effectiveness of pandemic vaccine during this period was significantly above zero (33%, 4–57), although not significant in most age subgroups except in the six to fourteen year-old group. Effectiveness of seasonal vaccines was, as for it, not significant, whether overall (19%, −10–41) or in each age subgroup. In conclusion, for both vaccine types, a moderate vaccine effectiveness was found after the 2009 A(H1N1) influenza virus stopped circulating at appreciable levels, which is indicative of upward biases in our VE estimates, but this post-pandemic effectiveness was mainly not statistically significant.
